# Modified hydrodissection for computed tomography-guided biopsy of mediastinal lesions: the “marshmallow” technique

**DOI:** 10.1590/0100-3984.2019.0010

**Published:** 2020

**Authors:** Chiang Jeng Tyng, Demian Jungklaus Travesso, Erich Frank Vater Santos, Almir Galvão Vieira Bitencourt, Paula Nicole Vieira Pinto Barbosa

**Affiliations:** 1 Department of Imaging, A.C.Camargo Cancer Center, São Paulo, SP, Brazil.

## INTRODUCTION

Computed tomography (CT)-guided percutaneous biopsies play a well-established role in the diagnosis, staging, and monitoring of cancer. These procedures are safe, effective, fast, affordable, and widely used in clinical practice. However, in some mediastinal lesions, percutaneous procedures may be challenging due to the proximity to vital structures and risk of severe complications. New techniques are continuously being developed to improve the approach to mediastinal lesions. Simple alternative methods (e.g., modifying the positioning of the patient, angling the gantry, using curved needles, and employing organ displacing techniques) or more complex methods (e.g., CT-fluoroscopy guidance with transsternal or transpulmonary access) have enabled an increasing number of procedures and have reduced the complication rates considerably^(1-3)^.

This study describes an innovative technique to perform CT-guided percutaneous biopsies of small mediastinal lesions located near vital structures. The new technique increases the applicability and safety of these procedures.

## TECHNIQUE

The “marshmallow” technique is essentially a variation of the hydrodissection technique, using sterile lidocaine gel instead of the traditional liquid material (saline/dextrose) to obtain a more effective and stable dissection of structures. This procedure is indicated in small mediastinal lesions located near vital structures, where an inadvertent/accidental puncture may have dramatic consequences. It is contraindicated in patients with hypersensitivity to lidocaine or its derivatives. It is also important to respect the lidocaine dose limit, including the amount used in superficial layers.

The use of a small amount of iodinated contrast provides greater contrast between the lesion and the adjacent structures, as well as allowing better control over the dispersion of the material. This was designated the “marshmallow” technique because the appearance of the lidocaine gel with iodinated contrast accumulated at the tip of the needle mimics that of a marshmallow on a stick.

**Figure 1:**
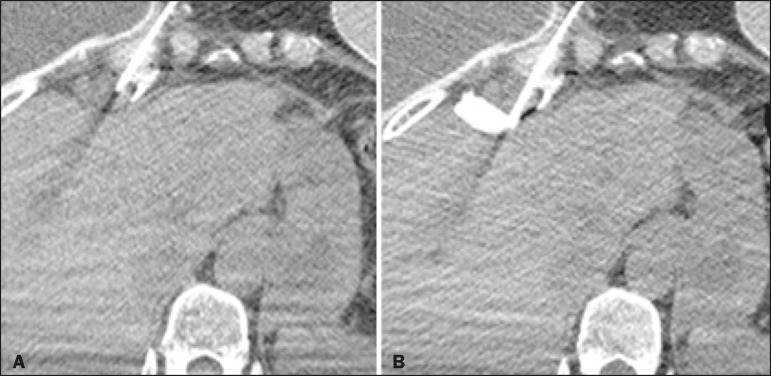
CT-guided percutaneous biopsy of a lymph node in the internal thoracic chain. **A:** Pre-procedure planning. **B:** Hydrodissection with lidocaine gel and iodinated contrast.

**Figure 2:**
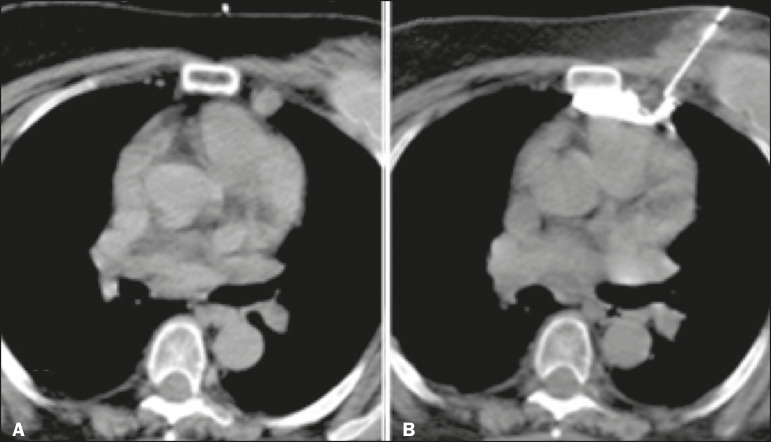
CT-guided percutaneous biopsy of a lesion in the right cardiophrenic space, adjacent to the liver. **A:** Coaxial needle positioned adjacent to the lesion. **B:** Hydrodissection with lidocaine gel and iodinated contrast after transfixation of the lesion with the coaxial needle.

The procedure consists in the following steps: patient positioning, monitoring, and sedation; imaging and planning; surgical scrub, including asepsis and sterile field placement; local anesthesia; coaxial needle placement (the coaxial needle should transfix the lesion, enabling the injection of lidocaine gel behind it); gel preparation; tissue dissection; and specimen retrieval. The radiologist prepares the gel by mixing 1 mL of iodinated contrast with 9 mL of lidocaine gel, using two syringes and a “T” connector, and inoculates the desired amount of the mixture, respecting the dose limit. Additional images are then acquired in order to verify the effectiveness of the dissection, after which the specimens can be retrieved (Figures 1 and 2).

## COMMENTS

Organ displacement techniques have recognized potential to create pathways, also known as “windows”, to access structures previously inaccessible to percutaneous procedures. Among these techniques, the most widespread and commonly used are artificial pneumothorax and hydrodissection^(3-5)^. The “marshmallow” technique is fundamentally a hydrodissection variant. The mixed liquid and solid properties inherent to lidocaine gel provide more effective and stable dispersion of the material, achieving satisfactory displacement of the adjacent structures. Through the transfixation of the lesion and subsequent inoculation of the gel, this technique provides an improvement in safety at the time of specimen retrieval, thus reducing the complication rates.

The “marshmallow” technique increases the applicability of percutaneous biopsies in mediastinal lesions, in which limited space and the high number of vital structures may complicate the execution of the procedure. Although its use is restricted to carefully selected cases, it may have great value, transforming complex, high-risk biopsies into safe, executable procedures.
